# Integration of clinical features and deep learning on pathology for the prediction of breast cancer recurrence assays and risk of recurrence

**DOI:** 10.1038/s41523-023-00530-5

**Published:** 2023-04-14

**Authors:** Frederick M. Howard, James Dolezal, Sara Kochanny, Galina Khramtsova, Jasmine Vickery, Andrew Srisuwananukorn, Anna Woodard, Nan Chen, Rita Nanda, Charles M. Perou, Olufunmilayo I. Olopade, Dezheng Huo, Alexander T. Pearson

**Affiliations:** 1 Department of Medicine, University of Chicago, Chicago, IL USA; 2 Department of Pathology, University of Chicago, Chicago, IL USA; 3 Tisch Cancer Institute, Icahn School of Medicine at Mount Sinai, New York, NY USA; 4 Department of Computer Science, University of Chicago, Chicago, IL USA; 5 Department of Genetics, Lineberger Comprehensive Cancer Center, The University of North Carolina at Chapel Hill, Chapel Hill, NC USA; 6 Department of Public Health Sciences, University of Chicago, Chicago, IL USA

**Keywords:** Breast cancer, Prognostic markers, Translational research

## Abstract

Gene expression-based recurrence assays are strongly recommended to guide the use of chemotherapy in hormone receptor-positive, HER2-negative breast cancer, but such testing is expensive, can contribute to delays in care, and may not be available in low-resource settings. Here, we describe the training and independent validation of a deep learning model that predicts recurrence assay result and risk of recurrence using both digital histology and clinical risk factors. We demonstrate that this approach outperforms an established clinical nomogram (area under the receiver operating characteristic curve of 0.83 versus 0.76 in an external validation cohort, *p* = 0.0005) and can identify a subset of patients with excellent prognoses who may not need further genomic testing.

Breast cancer is the leading cause of cancer death for women globally with an estimated 1.7 million cases diagnosed each year^1^. There is an unmet global clinical need for accurate diagnosis and treatment of breast cancer in response to the rising global burden of disease. Breast cancer is a biologically heterogeneous disease and genomic biomarkers have been developed to tailor therapeutic decisions. Hormone receptor-positive (HR + ) breast cancer constitutes about 70% of newly diagnosed cases in the United States^2^, although lower rates are generally seen outside of Western / European populations^3^. Gene expression-based recurrence score assays, such as OncotypeDx (ODX), MammaPrint (MP), Prosigna, and EndoPredict have been transformative for breast cancer management and are strongly recommended by the National Comprehensive Cancer Network^4^ and American Society of Clinical Oncology (ASCO)^5^ guidelines to aid decisions regarding the use of chemotherapy. However, genomic testing is costly^6^, is underutilized in minorities and low resource settings^7^, and can take weeks to perform leading to significant delays in care^8^. Clinical nomograms have been developed to identify patients at high risk of recurrence, but do not obviate the need for genomic testing^9^. Compared to gene expression assays, hematoxylin and eosin (H&E) stained pathology is readily available for all patients with cancer worldwide. Deep learning (DL) is a recent advance in the field of artificial intelligence (AI), which excels at quantitative image analysis. From histology, DL models can identify high-level image features, which in turn can be used to predict outcomes of interest, such as tumor grade, gene expression, and genetic alterations^10–12^. DL models trained on H&E pathology images have been shown to predict breast cancer gene expression, including molecular subtype as well as genes involved in cell cycle, angiogenesis, and immune response pathways^10,11,13^. Therefore, we hypothesized that a DL model incorporating digital pathology can outperform existing clinical models for the prediction of gene expression-based recurrence score assays.

To develop an accurate DL model for the prediction of recurrence score, we used a framework of two consecutive modules applied to image tiles extracted from the digital slide – one to predict tumor likelihood and a second to predict recurrence score results (Fig. 1a, b). The first DL module identified tumor regions of interest versus surrounding normal tissue using pathologist annotations from *n* = 1039 patients in The Cancer Genome Atlas (TCGA, Supplementary Table 1), achieving an average tile-level area under the receiver operating characteristic curve (AUROC) of 0.85 when assessed using internal three-fold cross-validation in TCGA. The second module was trained on image tiles from within the pathologist-annotated malignant areas from TCGA (*n* = 1039 patients) to predict the results of recurrence assays (calculated using gene expression data). A DL pathology recurrence score prediction was obtained by weighting the tile-level recurrence score by tile-level tumor likelihood across all tiles to provide a patient-level prediction. Furthermore, to assess if integrating clinical data improves the discriminatory capacity of our model, we developed a combined model incorporating the DL pathologic prediction and a clinical predictor of high ODX scores. A logistic regression was fit within TCGA-BRCA using our DL model prediction and ODX prediction from a previously published clinical nomogram developed by researchers from the University of Tennessee^9^. This clinical nomogram incorporates patient age, tumor size, progesterone receptor (PR) status, tumor grade, and histologic subtype. We tested this approach in the HR + /HER2- subset of our training cohort from TCGA (*n* = 535, Supplementary Table 1), reflective of the population where ODX is performed. Average AUROC for the prediction of high ODX score was 0.797 (95% CI 0.680–0.901) for the DL pathology model, 0.779 (95% CI 0.645–0.889) for the clinical nomogram, and 0.814 (95% CI 0.709–0.901) for the combined model (Fig. 1c).

**Fig. 1:**
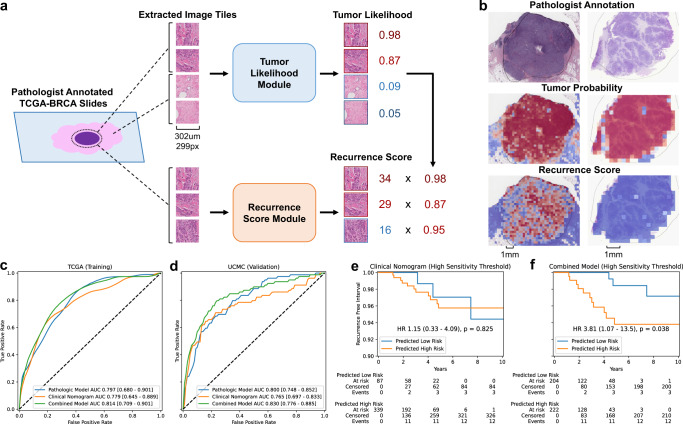
Overview of model architecture and results. **a** Xception-based deep learning models were trained on 1,039 patients from TCGA to allow for unsupervised predictions on external data. One model was trained to identify image tiles within pathologist annotation of tumor versus background image tiles (**b,** middle). The second model was trained to predict a research version of the 21-gene recurrence score calculated from gene expression data from the annotated tumor regions from TCGA (**b,** bottom). Finally, a combined clinical / pathologic model was developed by fitting a logistic regression to deep learning model predictions and the University of Tennessee clinical nomogram predictions. **c** Average patient-level AUROC for prediction of high-risk recurrence score in HR + /HER2- patients from TCGA on three-fold cross-validation (*n* = 535); the combined model AUROC was significantly higher than the clinical nomogram in two of three folds. **d** Patient-level AUROC for prediction of high-risk recurrence score in the UCMC cohort (*n* = 427); the combined model AUROC was significantly higher that the clinical nomogram in this cohort. **e, f** Kaplan-Meier curves illustrate recurrence-free interval in patients from the UCMC validation cohort predicted to have a high-risk Oncotype score using high-sensitivity thresholds (derived from TCGA) for each model. TCGA The Cancer Genome Atlas. AUC / AUROC Area Under the Receiver Operating Characteristic Curve, HR Hormone Receptor, UCMC University of Chicago Medical Center.

To validate these findings in an external cohort, we assessed the performance of the DL pathologic and combined models (frozen after training in TCGA) in *n* = 427 patients from the University of Chicago Medical Center (UCMC) who had ODX testing performed and pathologic samples available (Supplementary Table 2). AUROC for prediction of high ODX score of the combined model was 0.828 (95% CI 0.773–0.883), which was significantly higher than the clinical nomogram (AUROC 0.764, 95% CI 0.697–0.832, *p* = 0.0005) and a trend towards improvement over the DL pathologic model (AUROC 0.798, 95% CI 0.746–0.850, *p* = 0.155, Fig. 1d, Supplementary Table 3). Area under the precision-recall curve (AUPRC) was consistently highest for the combined model and exceeded random chance in all cases (Supplementary Table 3). The Spearman’s rank correlation coefficient between model predictions and numeric ODX score was consistently highest in the combined model (Supplementary Table 4, Supplementary Fig. 1). Performance was similar in Black and White patient subgroups (other racial/ethnic groups not assessed due to small sample size), with the combined model outperforming the clinical model in both subgroups (Supplementary Table 5). AUROCs remained highest with the combined model in several sensitivity analyses (Supplementary Table 6), however, the pathologic and thus the combined model performance declined when restricting training to HR + /HER2- patients in TCGA or when training on the smaller UCMC dataset, perhaps due to reduced training dataset size.

As ODX was initially developed to predict prognosis in patients treated with endocrine therapy alone, we evaluated the prognostic accuracy of models in patients treated without chemotherapy at UCMC (*n* = 322). We assessed prognostic accuracy using Cox regression, incorporating predictions from each model as a single variable. Each model was significantly associated with recurrence-free interval (RFI, Table 1), but the Harrell’s concordance index (C-index)^14^ was highest for the combined model (HR 2.04 per standard deviation, 95% CI 1.18–3.53, *p* = 0.011, C-index 0.743), nearly reaching the C-index of the actual ODX score (0.776). No model was associated with RFI among patients receiving chemotherapy, which may be due to confounding variables influencing treatment decisions and the use of ODX to select patients for treatment. Conversely, in TCGA, prognostic accuracy was highest for the clinical nomogram (C-Index 0.644, Supplementary Table 7), although no treatment information was available, and prognosis in this cohort was worse than expected for HR + /HER2- breast cancer receiving modern treatment regimens (Supplementary Fig. 2)^15,16^. Finally, we compared the ability of the three models to perform as highly-sensitive rule-out tests, to identify patients who do not require ODX testing. We selected a threshold for each model that achieved a sensitivity of 95% in the TCGA HR + /HER2- cohort (Supplementary Table 8), and then applied that threshold to UCMC patients. The true sensitivities were similar in the UCMC cohort, but the specificity was highest for the combined model (sensitivity 87.3%, specificity 55.2%) than for the Tennessee nomogram (sensitivity 88.6%, specificity 22.1%). Additionally, RFI was prolonged in patients deemed low-risk by the combined model using the high-sensitivity cut-off in both the TCGA (Supplementary Fig. 3) and UCMC validation cohort (Fig. 1e, f).

**Table 1:** Prognostic Value of Models in the Validation Cohort.

Endpoint	Model	C-Index, No CT	Hazard Ratio, No CT (95% CI)	z-statistic	*p* value	C-Index, CT	Hazard Ratio, CT (95% CI)	z-statistic	*p* value
RFI	Pathologic	0.707	1.72 (1.01–2.94)	1.98	0.048	0.5	0.9 (0.38–2.15)	−0.23	0.819
	Clinical	0.679	1.75 (1.09–2.81)	2.3	0.022	0.593	1.2 (0.56–2.57)	0.48	0.631
	Combined	0.743	1.55 (1.13–2.12)	2.74	0.006	0.513	1.02 (0.43–2.44)	0.05	0.962
	OncotypeDx	0.776	1.85 (1.32–2.59)	3.59	0.0003	0.628	1.43 (0.72–2.82)	1.03	0.305
RFS	Pathologic	0.561	1.1 (0.73–1.65)	0.46	0.646	0.532	0.82 (0.37–1.86)	−0.46	0.642
	Clinical	0.569	1.13 (0.76–1.68)	0.59	0.552	0.595	1.2 (0.59–2.43)	0.51	0.607
	Combined	0.574	1.17 (0.84–1.63)	0.93	0.354	0.498	0.95 (0.41–2.21)	−0.12	0.903
	OncotypeDx	0.647	1.45 (1.05–1.99)	2.26	0.024	0.636	1.4 (0.74–2.66)	1.02	0.306
OS	Pathologic	0.533	0.83 (0.49–1.4)	−0.7	0.484	0.529	0.78 (0.31–1.97)	−0.53	0.596
	Clinical	0.537	1.08 (0.67–1.74)	0.31	0.758	0.642	1.28 (0.56–2.92)	0.6	0.551
	Combined	0.489	1.06 (0.67–1.68)	0.23	0.816	0.481	0.93 (0.34–2.55)	−0.15	0.882
	OncotypeDx	0.581	1.33 (0.87–2.04)	1.33	0.183	0.694	1.41 (0.66–3.05)	0.88	0.377

*UCMC* University of Chicago Medical Center, *CT* Chemotherapy, *CI* Confidence Interval, *SD* Standard Deviation, *C-Index* Concordance Index, *RFI* Recurrence-free Interval, *RFS* Recurrence-free Survival, *OS* Overall Survival.

Results are listed for Cox proportional hazard models using the specified variable as the only input, for the subgroups of patients treated with (*n* = 103) or without chemotherapy (*n* = 322) in the UCMC validation cohort. Hazard ratios are computed per standard deviation of input data given the different scales of the various models, and results are given for the deep learning pathologic, clinical nomogram, and combined model along with OncotypeDx score as a gold standard comparator. Recurrence-free interval includes any recurrence events, whereas recurrence-free survival includes recurrence or survival events, and overall survival only includes survival events.

We performed a similar analysis to evaluate DL as a predictor of high-risk MP scores. As there is not a widely used nomogram for high-risk MP prediction, we developed a clinical predictor from the National Cancer Data Base (NCDB). The combined model had a trend towards higher accuracy in the prediction of high-risk MP scores (AUROC 0.759, 95% CI 0.656–0.861) than a clinical model (AUROC 0.741, 95% CI 0.634–0.849, *p* = 0.65) or a pathologic model (AUROC 0.739, 95% CI 0.633–0.846, *p* = 0.56) in a validation cohort of n = 88 UCMC patients (Supplementary Fig. 4, Supplementary Table 3). There was only one recurrence in the MP subgroup at UCMC, so prognostic comparisons to actual MP scores were not performed.

Finally, to help understand the nature of predictions made by this DL model, study pathologists independently reviewed heatmaps of the recurrence score module from 20 slides each with high-risk and low-risk ODX predictions in the UCMC cohort. Notable features identified by heatmaps included necrosis (both comedonecrosis and coagulative necrosis), lymphovascular invasion, high-grade nuclei, sheet-like growth of densely packed tumor nests, and infiltrative borders (Supplementary Fig. 5). The impact of tiles with pure necrosis and no visible tumor on model predictions was attuned by the fact that such tiles were also predicted to be non-cancer by our tumor likelihood model; however, tiles of tumor adjacent to necrosis may contribute predictions of high risk (Supplementary Fig. 6). To further demonstrate the correlation of these features with model predictions, we compared predictions in out-of-sample cases in the TCGA cohort with and without selecting previously annotated histologic features. We found that pathologic prediction of high-risk ODX was associated with higher grade (*p* = 1.76 × 10^−31^), lymphovascular invasion (*p* = 0.012), and necrosis (*p* = 1.52 ×10^−16^, Supplementary Table 9).

There are several prior attempts to use DL on pathologic images to improve the prediction of ODX scores. Two studies by Romo-Bucheli et al demonstrated that automated tubule nuclei quantification^17^ and mitotic activity^18^ can differentiate high versus low ODX scores; however, the reported accuracy analyses of these metrics excluded intermediate scores of 18–29 – limiting clinical applicability. Quantitative nuclear histomorphic features were found to have an AUROC of 0.65 in the identification of high ODX cases^19^ and a proprietary tile-based convolutional neural network model that deciphers cell, structure, and tissue-based features from image tiles was found to have an AUROC of 0.75 for prediction of high ODX^20^. The performance of our pathologic (AUROC of 0.80) and combined models (AUROC of 0.83) in the validation cohort may represent an advance over these approaches. DL models have been deployed incorporating clinical and immunohistochemical features scored by pathologists^21–23^, whereas our model only relies on universally available clinical parameters and H&E slide images. Strengths of this study include the consistency of performance for ODX prediction in both training and validation subsets, as well as in racial/ethnic subgroups (which is essential given potential inequities in DL^24^). Additionally, the correlation of predictions with known high-risk histologic factors, including grade, necrosis, and lymphovascular invasion suggest that biologically relevant features are identified by this weakly supervised DL pathologic approach. The high-sensitivity rule-out cutoff of the combined model identified a high proportion of patients as low risk in both datasets, and this cutoff was consistently prognostic for recurrence. However, there are limitations to our approach to model development. First, TCGA does not have clinical-grade recurrence assay results available, and a pathologic model trained and validated on clinical-grade assay results in this fashion may improve performance. Hyperparameter tuning in the TCGA cohort could lead to artificially inflated performance on internal validation, although reassuringly performance was preserved in the UCMC cohort. Our validation dataset had a low number of recurrence events, and the majority of survival events were from non-cancer mortality; thus larger sample sizes are needed to confirm the clinical utility of our approach. No model was prognostic in patients receiving chemotherapy and the prognostic value of the DL model was lower in TCGA. Although confounding factors may contribute to these findings, this further raises the need for confirmation of the prognostic value of the combined clinical / pathologic model. The magnitude of improvement of this deep learning approach over existing clinical models is small, but perhaps with additional training and refinement this approach can reach clinical-grade accuracy. Finally, it must be recognized that although the reported DL model was more accurate in identifying high-risk cases than a clinical nomogram, the true OncotypeDx recurrence assay had greater prognostic value than all evaluated models and remains the gold standard for treatment decisions in this population.

Understanding of the genomic features underlying cancer recurrence and chemotherapy benefit has evolved and genomic testing is now a routine part of breast cancer care. ASCO recently added the development and integration of deep learning technology into cancer research as a priority in 2021^25^, as artificial intelligence has the potential to rectify disparities and supplement or improve genomic testing. This study illustrates the development of an effective DL biomarker that improves on existing clinical predictors of low-recurrence risk tumors. ODX testing is estimated to grow in cost to $231 million annually in the USA^6^, and using a highly sensitive cutoff as described above could be used to limit testing in patients who are very unlikely to have positive results. Furthermore, given the heterogeneity of breast tumors, this methodology could be applied to multiple pathologic samples in a single patient to potentially increase confidence in results. With training on larger datasets with clinical-grade recurrence assays available to optimally tune thresholds, this approach could improve the speed at which treatment decisions are made due to the time-consuming nature of genomic testing, reduce the cost of care, and be utilized worldwide where genomic assays are not available.

## Methods

### Ethics statement

All experiments were conducted in accordance with the Declaration of Helsinki and the study was approved by the University of Chicago Institutional Review Board, IRB 22-0707. For model training, patients were included from the TCGA breast cancer cohort (BRCA)^26^. For validation, anonymized archival tissue samples were retrieved from the University of Chicago from January 1st 2006, through December 21st 2020, where recurrence score results were available. Informed consent for this study was waived, as patients had previously consented to the secondary use of their biospecimens.

### Model Development

First, an automated tumor detection module was trained to distinguish breast tumor from background tissue in digitally scanned H&E slides. From TCGA, 1133 slides were reviewed, and 1,106 from 1,046 patients had acceptable quality tumor-rich regions identified on pathologist review. Seven slides had encoding errors preventing processing in our pipeline, leaving us with a cohort of 1,099 slides from 1039 patients, which were annotated manually by study pathologists to distinguish tumor from surrounding stroma. Tessellated image tiles were extracted from within areas of tumor with an edge length of 302 microns and downscaled to a width of 299 pixels, consistent with an optical resolution of 10x. Tile extraction and DL model training was performed with the Slideflow pipeline^27^, using an Xception^28^ convolutional neural network backbone pretrained on ImageNet and with all layers fine-tuned during training, with a variable number of fully connected hidden layers prior to outcome prediction. The tumor likelihood module was trained with hyperparameters as listed in Supplementary Table 10 to distinguish tiles originating from within the tumor annotation from those outside the annotation. Model performance was assessed with average accuracy over three cross-fold validation, and a separate model was trained on the entire dataset for prediction on external patients. The flow of data used for hyperparameter optimization, model training, and validation is illustrated in Supplementary Fig. 7.

Next, a separate DL module was trained to predict recurrence score from tumor image tiles extracted from the pathologist-annotated region of interest. As the clinically validated multigene recurrence assay results are not available from TCGA, “research-based” versions of ODX and MP were calculated using upper quantile normalized star-salmon gene-level expression data from TCGA. Sequencing data was log (base 2) transformed and row median centered and column standardized across TCGA-BRCA. Statistical formulas from the published development of OncotypeDx^29^ and MammaPrint^30,31^ were then applied to the mRNA expression data to calculate research-based recurrence scores.

This module is trained in a weakly supervised fashion, with the results of the patient-level mRNA assay assigned to each tumor tile. To determine a threshold for high-risk “research-based” ODX score, the 15^th^ percentile result of HR + /HER2- patients in TCGA was used, as this is the percentile of patients with ODX score of 26 or higher in the National Cancer Database^9^. TCGA model training was not restricted to HR + /HER2- patients to enrich for samples with high-risk ODX predictions, but internal validation in TCGA was performed in the HR + /HER2- subset. In the UCMC, we used standard high-risk cutpoints of ODX score of 26 or higher, and MP score of lower than 0. Hyperparameters for these models were chosen with Bayesian optimization of cross-validated tile level AUROC, run over 50 iterations (Supplementary Table 10, Supplementary Fig. 8). Two sets of three cross folds were used for optimization, and although samples from TCGA were H&E stained at a single site, folds were generated with site preservation^32^ to maximize generalizability given prior reports of site-specific batch effect present in TCGA. Patient-level predictions were calculated by weighting the average of tile-level predictions from this recurrence score prediction module according to a tile’s likelihood of tumor from the first module. Thus, all extracted tiles (after grayspace filtering) contributed to model predictions.

For clinical prediction of recurrence the University of Tennessee Nomogram^9^ was computed for each patient in TCGA; grade is not available in the original TCGA annotations but has been assessed and reported in prior work^33^. Precise tumor size was not provided in TCGA but was estimated from tumor stage group, and mean imputation was used in TCGA for three cases where progesterone receptor status was not available for nomogram estimation – no imputation was needed for nomogram calculation in the UCMC dataset. Finally, logistic regression models were fit using the out-of-sample prediction from the pathologic model combined with the prediction from the clinical nomogram, and then validated in held-out data from TCGA. The coefficients of the logistic regressions fit in TCGA were averaged to define the model used for external validation. Thresholds for computing model sensitivity were determined from TCGA (using interpolation to achieve an exact estimated sensitivity of 95%) and applied to the validation dataset from UCMC.

Development of the MP prediction model proceeded in a similar fashion with a few key differences. As no widely used clinical model was available, we developed a clinical predictor from *n* = 6,938 nonmetastatic HR + /HER2- patients from NCDB who were diagnosed with breast cancer between 2010 and 2017 and had MP testing results available. We used sequential forward feature selection to identify features that improved the AUROC for MP prediction in a logistic regression with 10-fold cross-validation, ultimately identifying grade, tumor size, PR status, lymphovascular invasion, ductal, mucinous, metaplastic, or medullary histology, and Black or Asian race for inclusion. A logistic regression incorporating these features was fit on all available data and used for prediction. We used the same optimized hyperparameters from ODX prediction for our DL pathologic MP model.

### Statistical analysis

Internal validation of model accuracy for recurrence score prediction in TCGA was estimated by averaging patient-level AUROC and AUPRC over three-fold site-preserved cross-validation, and 1000x bootstrapping for confidence interval estimation. External validation was performed with single fixed models generated from all TCGA data, using Delong’s method for statistical comparison of AUROCs^34^. The prognostic accuracy of models for RFI was assessed with the Wald test in univariable Cox models. Two-sided t-tests were performed to compare DL pathologic model predictions between patients with or without select pathologic features. All statistical analysis was performed in Python 3.8, Lifelines 0.27.0, and Scipy 1.8.0 and performed at the α = 0.05 significance level. Given the limited number of statistical tests, performed in different subsets of patients, and the exploratory nature of this work, correction for multiple hypothesis testing was not performed.

### Reporting summary

Further information on research design is available in the Nature Research Reporting Summary linked to this article.

## Supplementary information

Supplemental Materials

Reporting Summary

## Data Availability

Data from TCGA including digital histology and the clinical and genetic annotations used are available from https://portal.gdc.cancer.gov/ and https://cbioportal.org, and the annotations used for grade, necrosis, and lymphovascular invasion are from previously published work^33^. The NCDB PUF is a HIPAA-compliant data file, which is made available to investigators from CoC-accredited cancer programs who complete an application process. Trained models evaluated in this paper, anonymized patient annotations, and the complete set of tile images used for model validation can be obtained at 10.5281/zenodo.7490381.

## Code availability

Code utilized in model development and assessment is available at https://github.com/fmhoward/DLRS.
