# Supplementary material for: Integration of clinical features and deep learning on pathology for the prediction of breast cancer recurrence assays and risk of recurrence
Source: NPJ Breast Cancer. 2023 Apr 14;9:25. doi: 10.1038/s41523-023-00530-5 (PMC10104799; doi:10.1038/s41523-023-00530-5)
Supplement: Supplementary file 1 — Supplemental Materials [file 41523_2023_530_MOESM1_ESM.pdf]

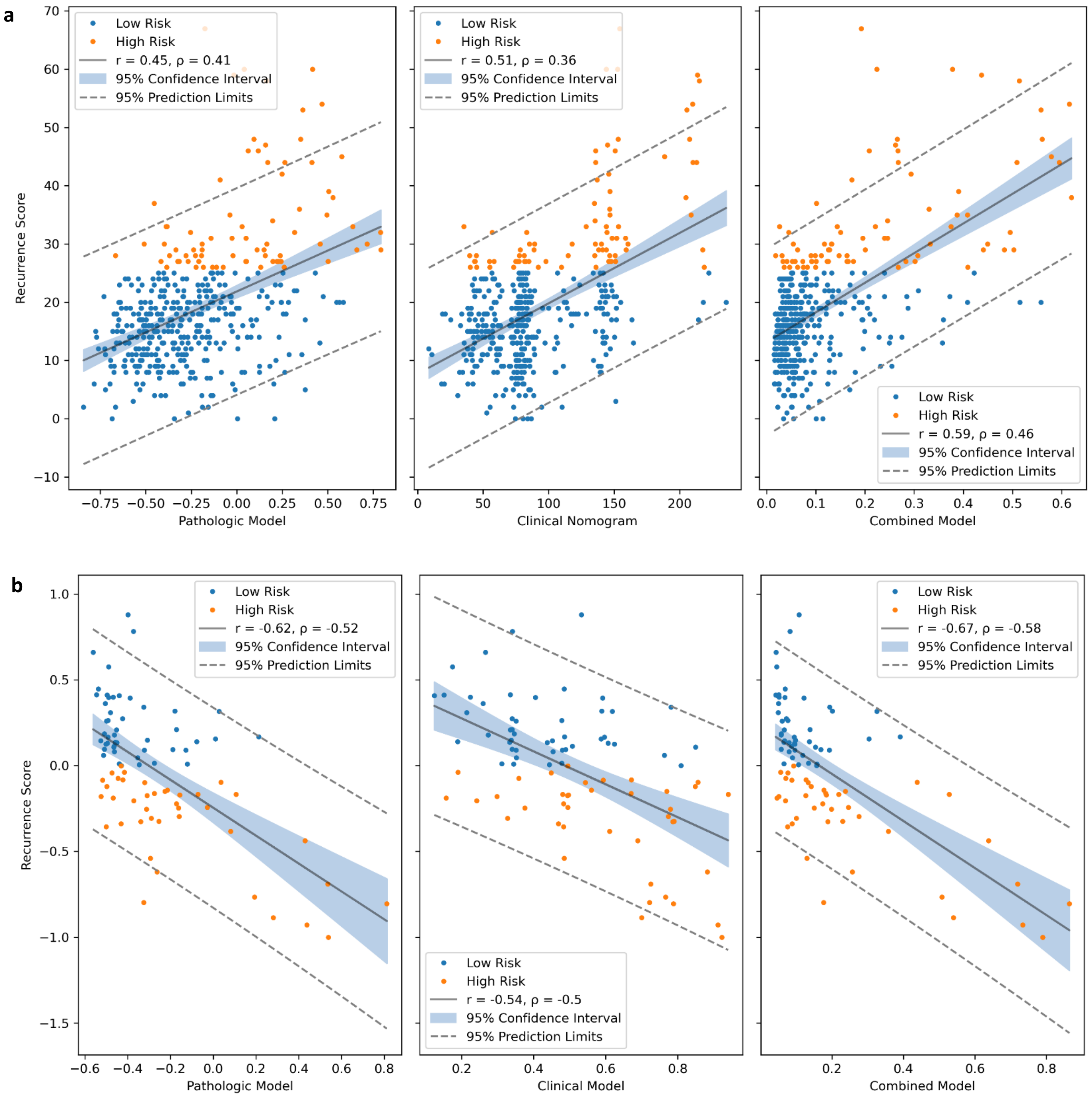

Supplementary Figure 1. Linear Correlation of Models Predictions with OncotypeDx and MammaPrint Scores. a. Correlation of the deep learning pathology, Tennessee clinical nomogram, and combined model with OncotypeDx scores in the UCMC cohort (n = 427). Pearson's correlation coefficient (r) is reported, and given skew present in the distribution, Spearman's rank correlation coefficient (ρ) is also reported. **b**. Correlation of the deep learning pathology, clinical, and combined model with MammaPrint scores in the UCMC cohort (n = 88). **Abbreviations: UCMC = University of Chicago Medical Center.**

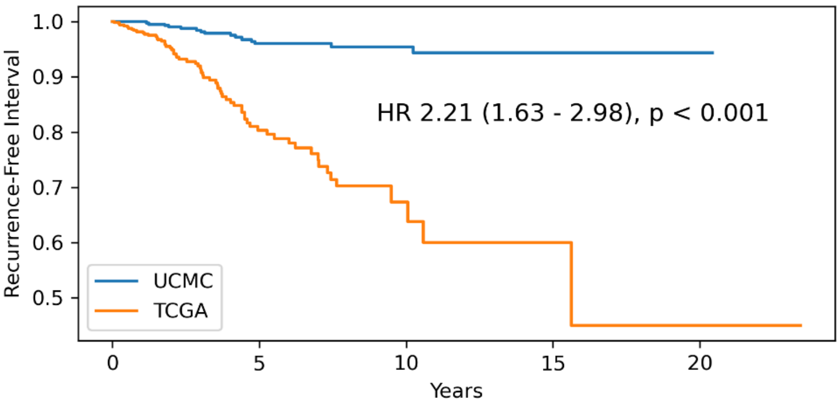

**UCMC**					
At risk	425	250	91	6	1
Censored	0	162	320	404	409
Events	0	13	14	15	15
**TCGA**					
At risk	527	119	20	4	2
Censored	8	365	453	467	468
Events	0	51	62	64	65

Supplementary Figure 2. Recurrence Free Interval for the UCMC and TCGA Cohorts. Survival analysis reveals a highly significant difference in recurrence-free interval between the patients in the training (TCGA) and validation (UCMC) cohorts. **Abbreviations: TCGA = The Cancer Genome Atlas. UCMC = University of Chicago Medical Center.**

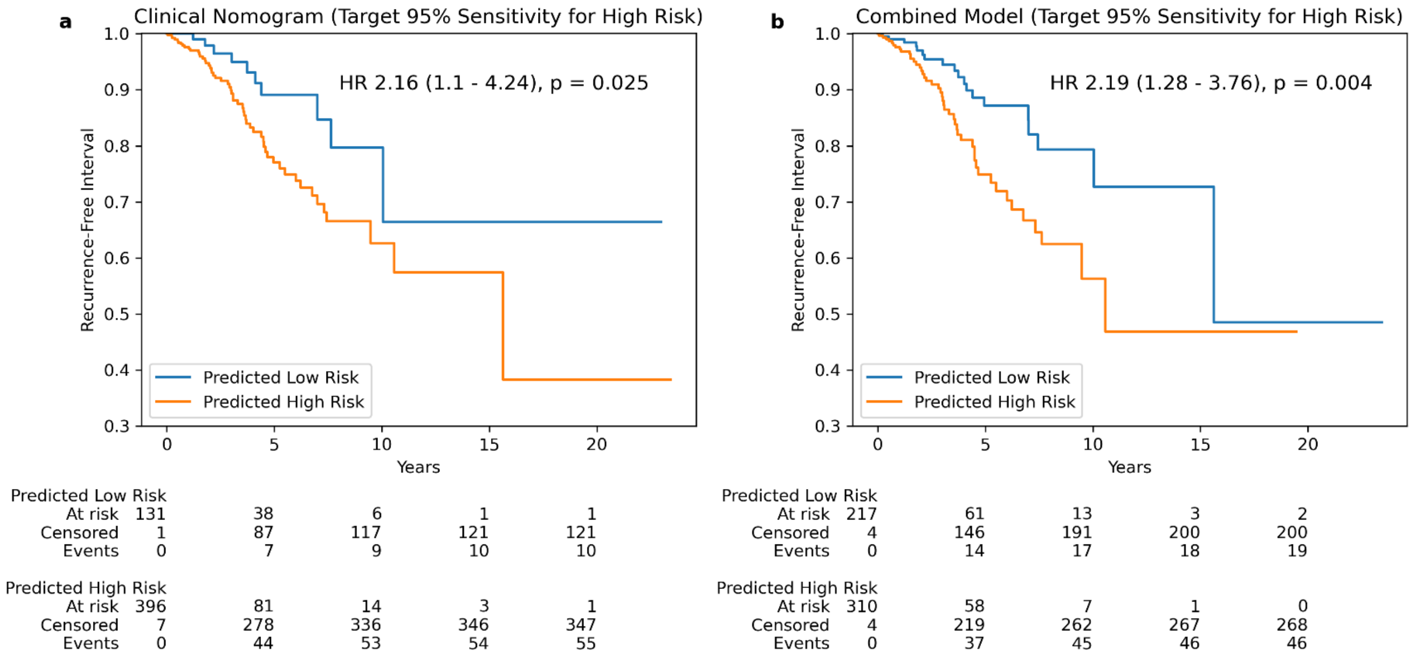

Supplementary Figure 3. Prognostic Value of a High Sensitivity Rule-Out Threshold in the Training Dataset. We assessed the performance of a high-sensitivity rule-out threshold for our models in the TCGA training cohort (n = 535) prior to external validation, by examining the accuracy for prediction of recurrence-free interval. The combined clinical / pathologic model threshold was prognostic for recurrence with a greater proportion classified as low risk, serving as a more effective rule-out test, although the hazard ratios for recurrence were similar.

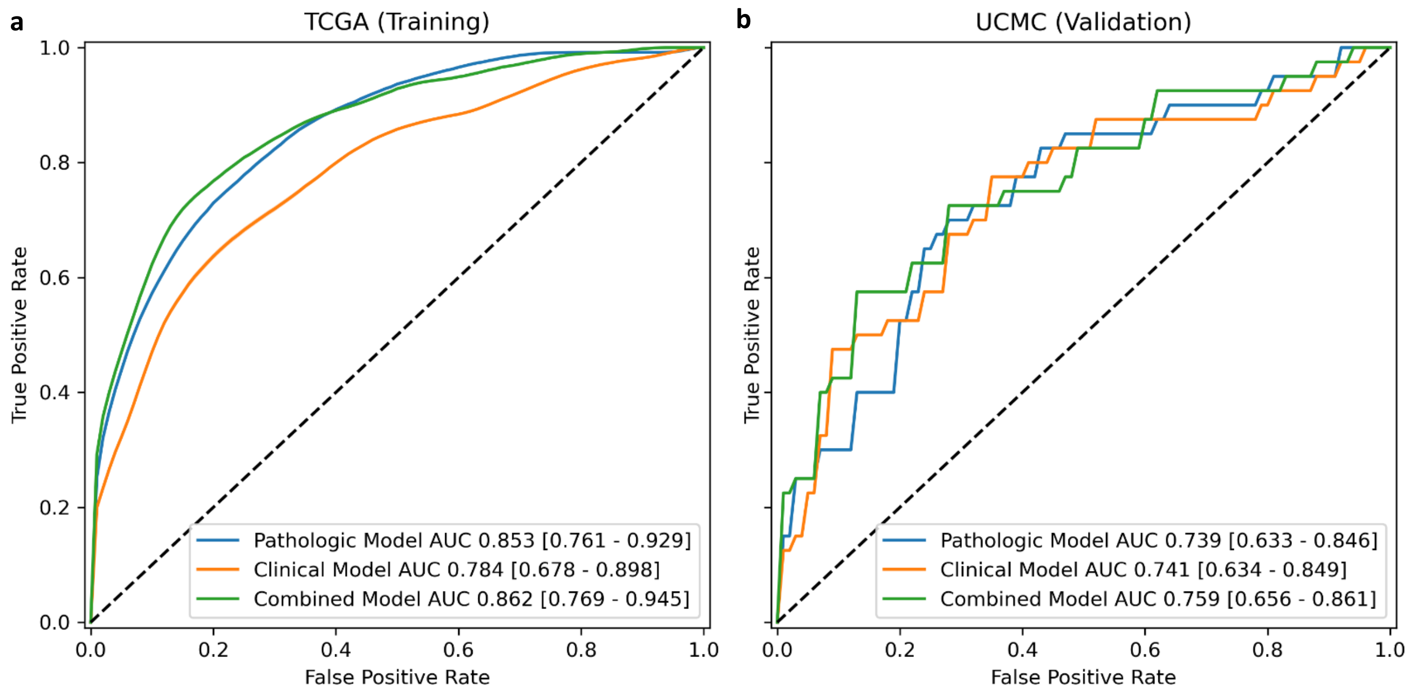

Supplementary Figure 4. Predictive Accuracy of MammaPrint Prediction Models. a. Receiver operating characteristic curves for MammaPrint prediction for the deep learning pathologic, clinical model, and combined models in HR+/HER2- patients from TCGA (n = 535). **b.** The same curves plotted for the external UCMC cohort (n = 88). **Abbreviations: TCGA = The Cancer Genome Atlas. UCMC = University of Chicago Medical Center.**

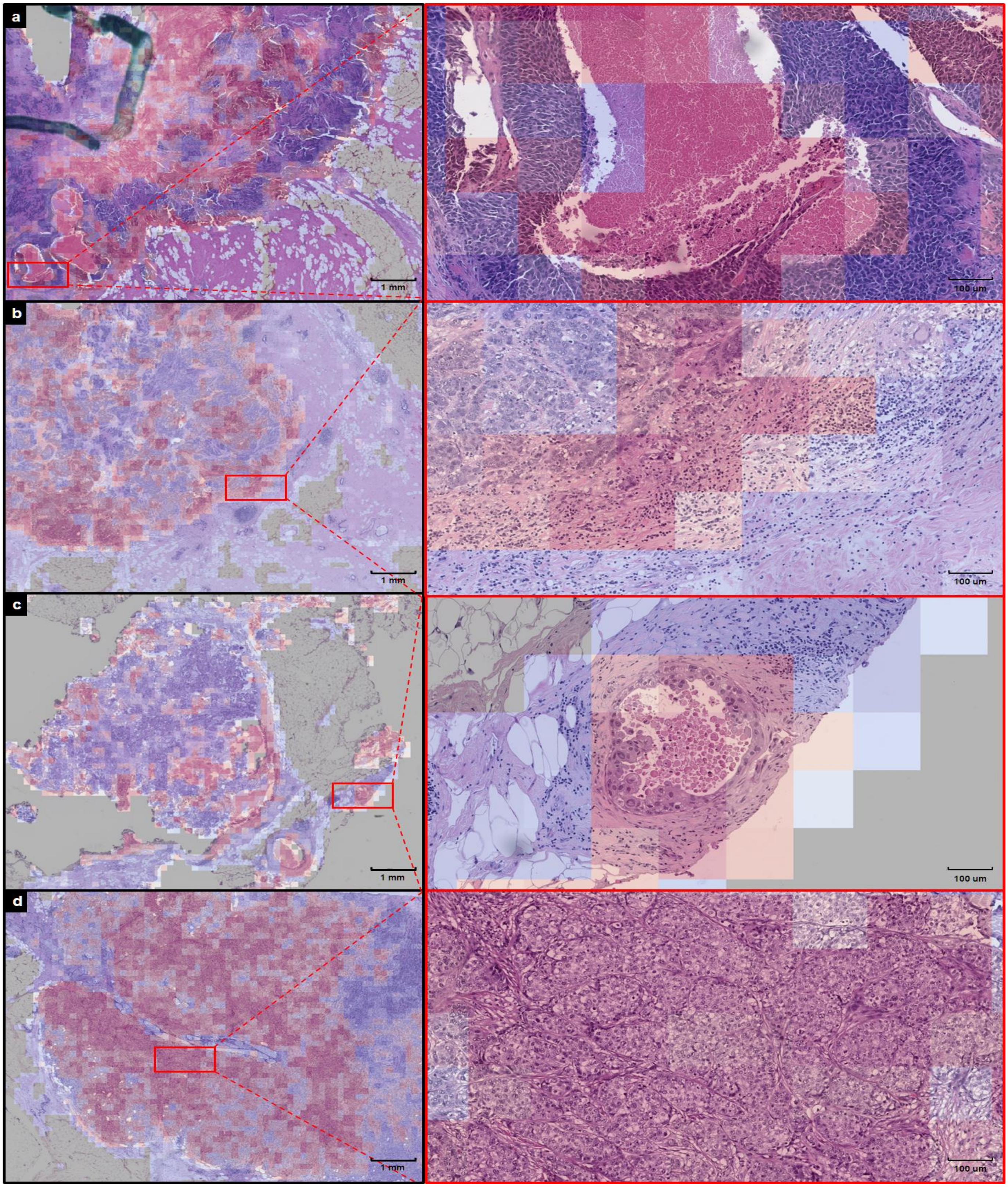

Supplementary Figure 5. Heatmaps of the Recurrence Score Prediction Model on Select High-Risk Tumors. Review of patients with predicted high recurrence scores in the validation dataset identified several pathologic characteristics shared between tumors with high-risk predictions, including **a.** Comedo necrosis, **b.** infiltrative borders, **c.** lymphovascular invasion, and **d.** high grade densely packed tumor nests.

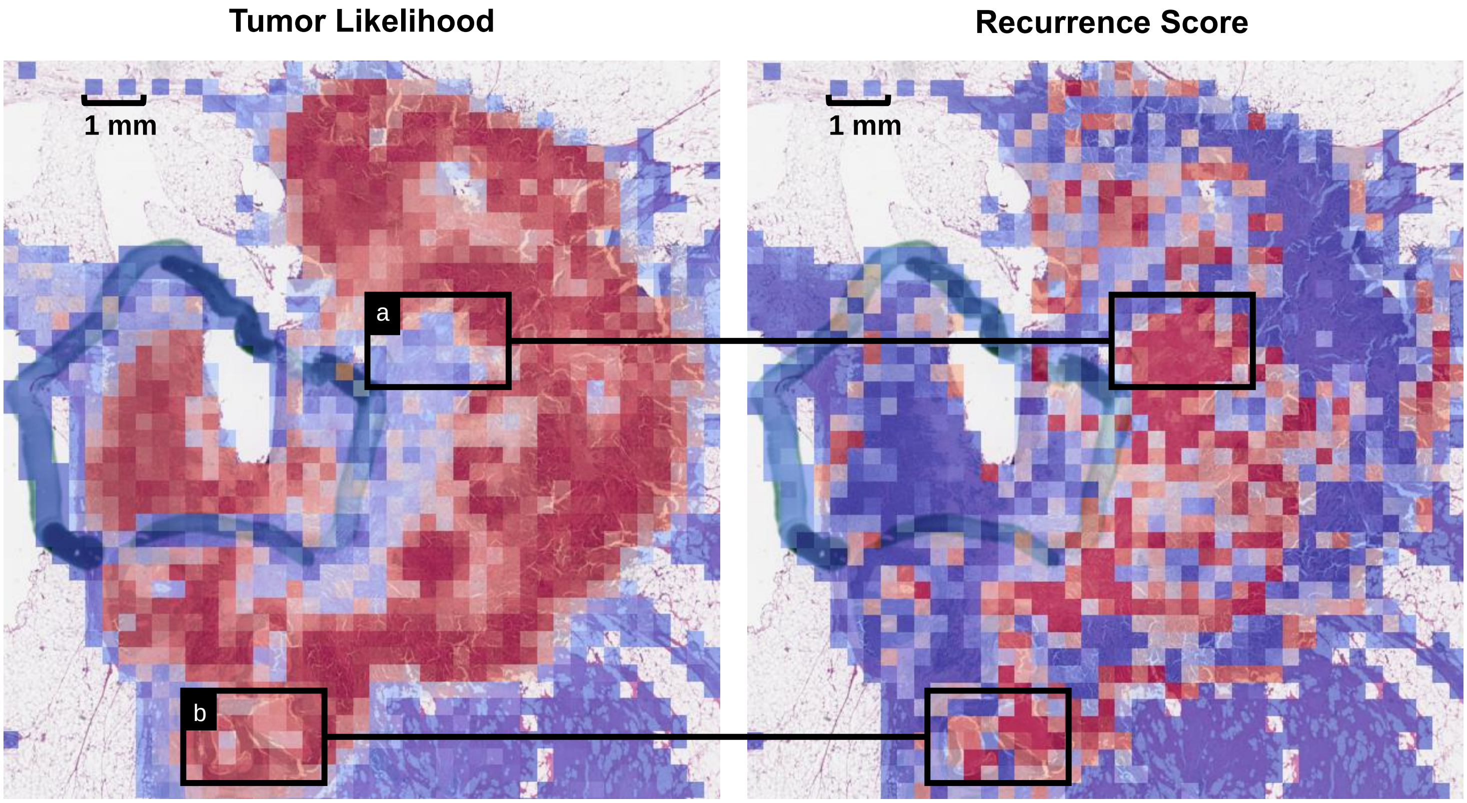

Supplementary Figure 6. Interplay Between Tumor Likelihood and Recurrence Score Modules. Review of a patient with prominent necrosis on biopsy sample and high predicted recurrence score. Both coagulative necrosis (**a**) and comedo type necrosis (**b**) demonstrate lower likelihood of tumor and high recurrence score prediction. However, the tumor likelihood value is high at the interface of tumor and necrosis, providing a mechanism for the correlation of model prediction for recurrence score and presence of necrosis.

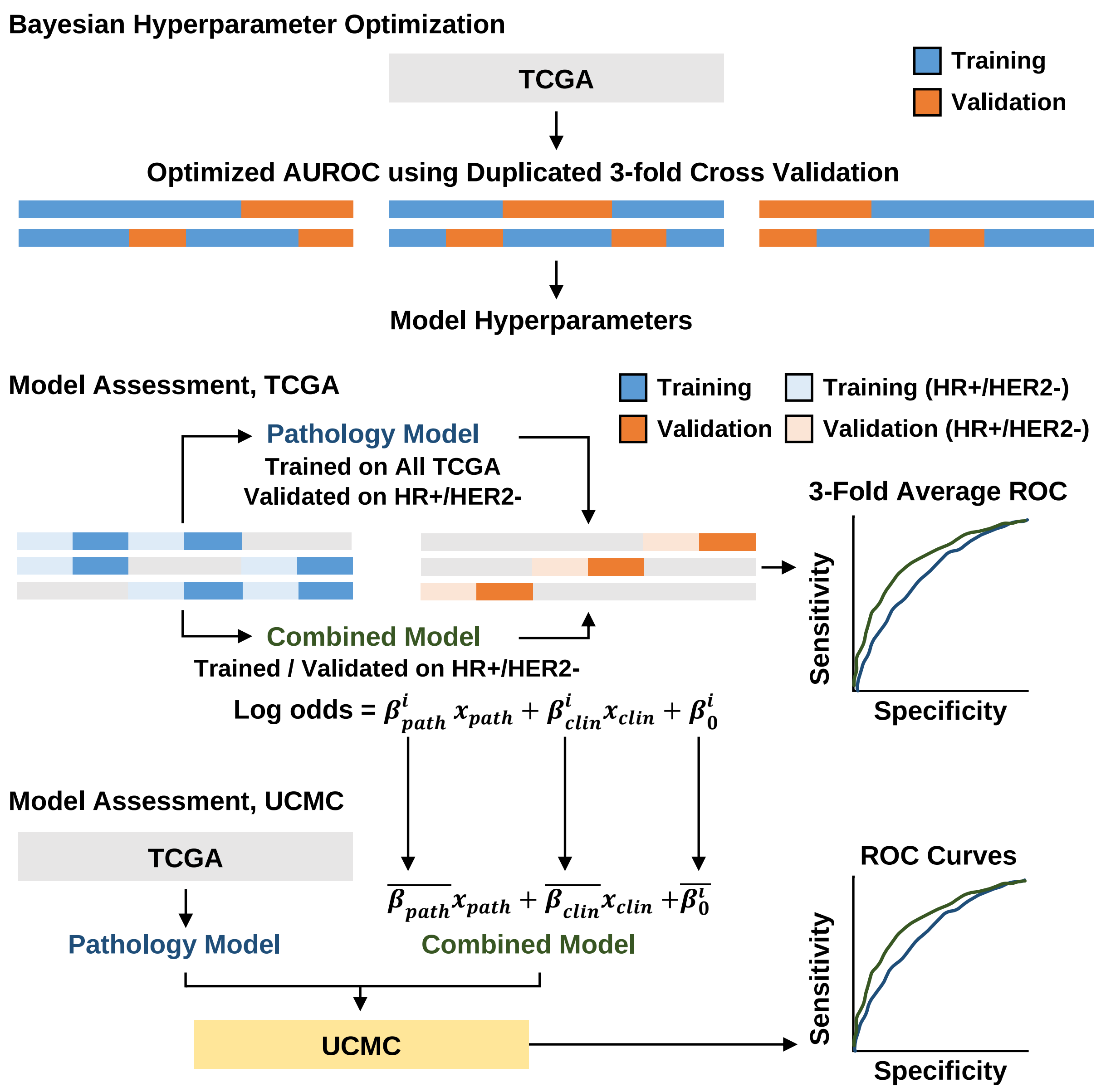

Supplementary Figure 7. Schema for Data Use for Hyperparameter Selection, Model Training, and Assessment. The entire TCGA dataset (n = 1,039) was used for Bayesian hyperparameter optimization, using two sets of site preserved splits. The average AUROC for genomic assay result was calculated in the held-out third of the data for each of these six splits and used for hyperparameter optimization. Pathology models were then trained on TCGA (n = 1,039) inclusive of all cancer subtypes to optimize detection of high risk features in HR- and HER2+ cancers, with performance estimated with three-fold preserved site cross validation in the HR+/HER2- cohort (n = 535). Three combined (clinical + pathologic) models were fit using held-out pathology predictions on HR+/HER2- patients from these same cross folds, and subsequently validated in HR+/HER2- patients. Average performance for these three pathology and combined models are reported. For prediction in

the UCMC dataset, a pathology model was trained using the full TCGA dataset, and the combined logisitc regression model was formulated using average coefficients from the three logistic regression models trained in TCGA. **Abbreviations: TCGA = The Cancer Genome Atlas. AUROC = Area Under the Receiver Operating Characteristic Curve. ROC = Receiver Operating Characteristic. UCMC = University of Chicago Medical Center.**

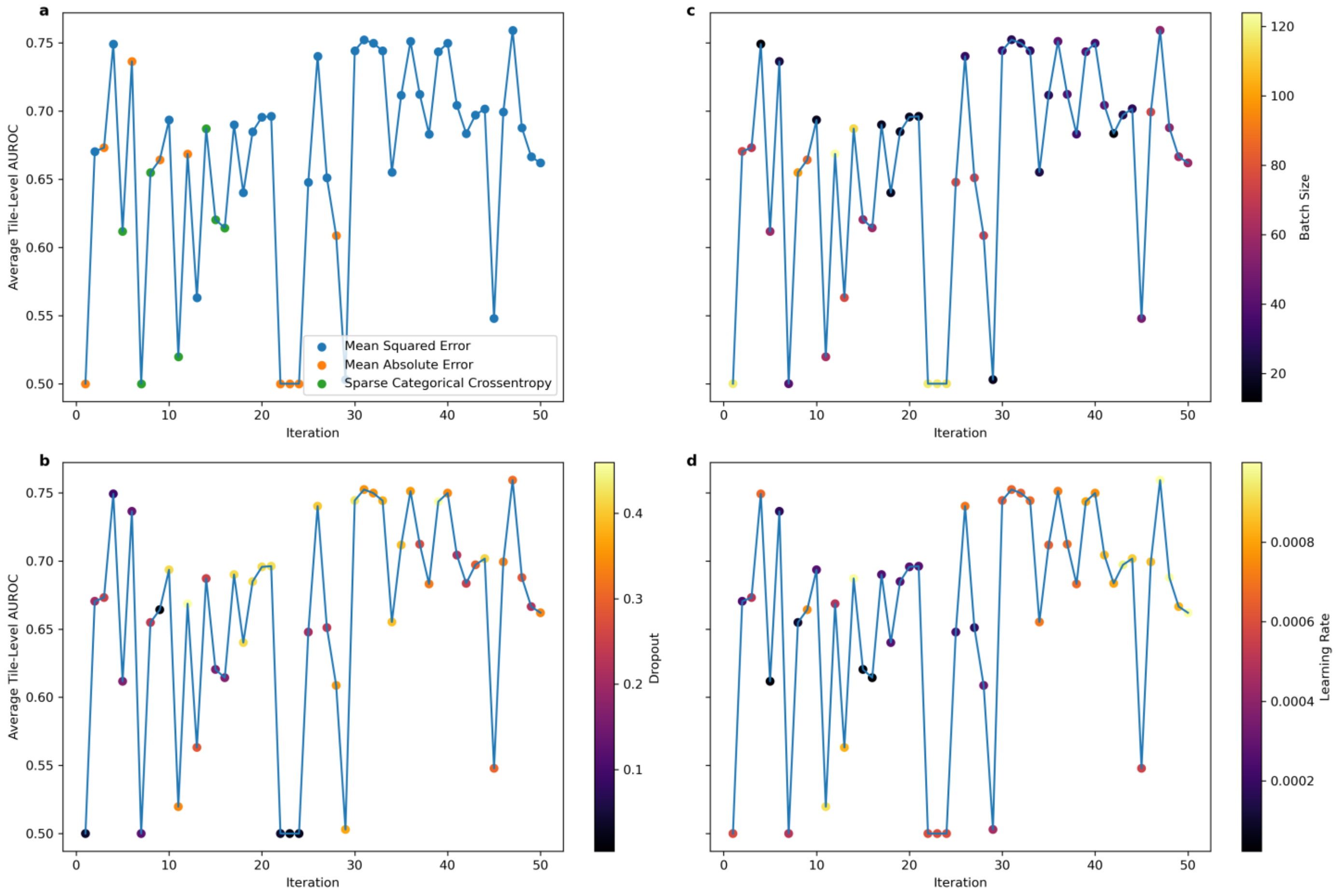

Supplementary Figure 8. Tile-Level AUROC for Prediction of High-Risk OncotypeDx Score During Hyperparameter Optimization. Results are listed over 50 iterations of Bayesian optimization with objective of maximizing average tile-level AUROC over two replicates of three site-preserved cross folds in The Cancer Genome Atlas. Plots highlight the associations of selected hyperparameters **a.** loss function, **b.** batch size, **c.** dropout, and **d.** learning rate with model performance. **Abbreviations: AUROC = Area Under the Receiver Operating Characteristic Curve.**

Supplementary Table 1. Baseline Demographics from Cohorts from The Cancer Genome Atlas used for Model Training. Abbreviations: HR+ = Hormone Receptor Positive. HER2 = Human Epidermal Growth Factor Receptor 2. SD = Standard Deviation. ER = Estrogen Receptor. PR = Progesterone Receptor.

		Missing	Overall	Missing	HR+/HER2-
n			1039		535
Age, mean (SD)		0	58.6 (13.2)	0	59.2 (13.2)
Sex, n (%)	Female	0	1027 (98.8)	0	531 (99.3)
	Male		12 (1.2)		4 (0.7)
Race, n (%)	Asian	94	60 (6.3)	62	23 (4.9)
	Black		162 (17.1)		45 (9.5)
	Other		1 (0.1)		0 (0.0)
	White		722 (76.4)		405 (85.6)
Ethnicity, n (%)	Hispanic	166	38 (4.4)	111	18 (4.2)
	Non-Hispanic		835 (95.6)		406 (95.8)
Histologic Subtype, n (%)	Ductal	0	632 (60.8)	0	314 (58.7)
	Ductal and Lobular		114 (11.0)		63 (11.8)
	Lobular		176 (16.9)		93 (17.4)
	Other		117 (11.3)		65 (12.1)
Grade, n (%)	1	2	229 (22.1)	0	165 (30.8)
	2		428 (41.3)		241 (45.0)
	3		380 (36.6)		129 (24.1)
Tumor Size (mm) inferred from T stage, mean (SD)		0	31.8 (12.1)	0	31.6 (12.3)
Nodal Status, n (%)	Negative	19	488 (47.8)	8	251 (47.6)
	Positive		532 (52.2)		276 (52.4)
ER Status, n (%)	Negative	49	224 (22.6)	0	13 (2.4)
	Positive		766 (77.4)		522 (97.6)
PR Status, n (%)	Negative	52	325 (32.9)	3	68 (12.8)
	Positive		662 (67.1)		464 (87.2)
HER2 Status, n (%)	Negative	195	697 (82.6)	0	522 (100.0)
	Positive		147 (17.4)		0 (0.0
Research-Only OncotypeDx Score, mean (SD)		0	0.1 (1.0)	0	-0.3 (0.8)
Research-Only MammaPrint Score, mean (SD)		0	-0.0 (1.0)	0	0.3 (0.8)
Follow-up, mean (SD)		1	3.5 (3.3)	0	3.6 (3.3)
Recurrence, n (%)	Disease Free	0	902 (86.8)	0	470 (87.9)
	Recurred		137 (13.2)		65 (12.1)
Vital Status, n (%)	Alive	0	893 (85.9)	0	467 (87.3)
	Dead		146 (14.1)		68 (12.7)

Supplementary Table 2. Baseline Demographics from Cohorts from University of Chicago Medical Center used for Model Validation. Abbreviations: ER = Estrogen Receptor. PR = Progesterone Receptor. HER2 = Human Epidermal Growth Factor Receptor 2. SD = Standard Deviation.

		Missing	OncotypeDx Cohort	Missing	MammaPrint Cohort
**n**		-	427	-	88
**Age, mean (SD)**		0	56.3 (10.6)	0	54.0 (12.7)
**Sex, n (%)**	**Female**	0	423 (99.1)	0	88 (100.0)
	**Male**		4 (0.9)		0 (0.0)
**Race, n (%)**	**Asian**	8	27 (6.4)	1	3 (3.4)
	**Black**		102 (24.3)		23 (26.4)
	**Other**		2 (0.5)		1 (1.1)
	**White**		288 (68.7)		60 (69.0)
**Ethnicity, n (%)**	**Hispanic**	5	13 (3.1)	2	5 (5.8)
	**Non-Hispanic**		409 (96.9)		81 (94.2)
**Histologic Subtype, n (%)**	**Ductal**	0	302 (70.7)	0	68 (77.3)
	**Ductal and Lobular**		40 (9.4)		9 (10.2)
	**Lobular**		71 (16.6)		10 (11.4)
	**Other**		14 (3.3)		1 (1.1)
**Grade, n (%)**	**1**	0	68 (15.9)	0	10 (11.4)
	**2**		279 (65.3)		58 (65.9)
	**3**		80 (18.7)		20 (22.7)
**Tumor Size (mm), mean (SD)**		0	21.0 (16.2)	0	25.5 (23.1)
**Nodal Status, n (%)**	**Negative**	0	362 (84.8)	0	15 (17.0)
	**Positive**		65 (15.2)		73 (83.0)
**ER Status, n (%)**	**Negative**	1	9 (2.1)	0	7 (8.0)
	**Positive**		417 (97.9)		81 (92.0)
**PR Status, n (%)**	**Negative**	0	53 (12.4)	0	12 (13.6)
	**Positive**		374 (87.6)		76 (86.4)
**HER2 Status, n (%)**	**Negative**	9	410 (98.1)	4	83 (98.8)
	**Positive**		8 (1.9)		1 (1.2)
**Recurrence Score (OncotypeDx or MammaPrint), mean (SD)**		0	18.6 (10.1)	0	-0.0 (0.4)
**Chemotherapy, n (%)**	**No**	0	323 (75.6)	0	49 (55.7)
	**Yes**		104 (24.4)		39 (44.3)
**Years Follow-up, mean (SD)**		0	6.8 (4.0)	0	3.2 (2.4)
**Recurrence, n (%)**	**Disease Free**	2	410 (96.5)	3	84 (98.8)
	**Recurred**		15 (3.5)		1 (1.2)
**Vital Status, n (%)**	**Alive**	0	407 (95.3)	0	86 (100.0)
	**Dead**		20 (4.7)		0 (0.0)

Supplementary Table 3. Predictive Accuracy for Recurrence Score. Results are listed for correlation with numeric recurrence score, and AUROC for prediction of high-risk recurrence score with 95% confidence intervals generated with 1000x bootstrapping. For the validation dataset, the AUROC of the combined model was compared to the individual component pathologic / clinical models using Delong's method. Given the imbalanced rate of high-risk recurrence score results, AUROC can be artificially inflated, so we also computed AUPRC and 95% confidence intervals. Expected AUPRC with random chance was 0.136 and 0.185 for the training and validation cohorts for the OncotypeDx models, and 0.204 and 0.460 for the training and validation cohorts of the MammaPrint models respectively. **Abbreviations: AUROC = Area Under the Receiver Operating Characteristic Curve. AUPRC = Area Under the Precision Recall Curve. TCGA = The Cancer Genome Atlas. UCMC = University of Chicago Medical Center. NCDB = National Cancer Database.**

	**TCGA (Training)**		**UCMC (Validation)**			
					**AUROC Compared to Combined Model**	
	**AUROC**	**AUPRC**	**AUROC**	**AUPRC**	**z-statistic**	**p – value**
			**OncotypeDx**			
**Pathologic Model**	0.797 (0.680 - 0.901)	0.401 (0.194 - 0.627)	0.798 (0.746 - 0.850)	0.501 (0.383 - 0.617)	1.42	0.15
**Clinical Nomogram**	0.779 (0.645 - 0.889)	0.396 (0.147 - 0.671)	0.764 (0.697 - 0.832)	0.486 (0.372 - 0.609)	3.49	0.0005
**Combined Model**	0.814 (0.709 - 0.901)	0.452 (0.202 - 0.691)	0.828 (0.773 - 0.883)	0.599 (0.477 - 0.703)	-	-
			**MammaPrint**			
**Pathologic Model**	0.853 (0.761 - 0.929)	0.642 (0.456 - 0.811)	0.739 (0.633 - 0.846)	0.722 (0.560 - 0.844)	0.58	0.56
**Clinical Model (from NCDB)**	0.784 (0.678 - 0.898)	0.564 (0.371 - 0.742)	0.741 (0.634 - 0.849)	0.721 (0.572 - 0.850)	0.45	0.65
**Combined Model**	0.862 (0.769 - 0.945)	0.682 (0.486 - 0.845)	0.759 (0.656 - 0.861)	0.757 (0.623 - 0.867)	-	-

Supplementary Table 4. Correlation of Model Predictions with True Recurrence Score. Results are listed for Pearson's correlation coefficient and Spearman's rank correlation coefficient with numeric recurrence score for the OncotypeDx and MammaPrint models in both the training and validation cohort. **Abbreviations: TCGA = The Cancer Genome Atlas. UCMC = University of Chicago Medical Center. AUROC = Area Under the Receiver Operating Characteristic Curve. NCDB = National Cancer Database.**

	**TCGA (Training)**		**UCMC (Validation)**	
	**Pearson's Correlation Coefficient**	**Spearman's Rank Correlation Coefficient**	**Pearson's Correlation Coefficient**	**Spearman's Rank Correlation Coefficient**
**OncotypeDx**				
**Pathologic Model**	0.557 (0.495 - 0.613)	0.545 (0.483 - 0.602)	0.446 (0.367 - 0.519)	0.407 (0.324 - 0.483)
**Clinical Nomogram**	0.539 (0.476 - 0.597)	0.519 (0.454 - 0.578)	0.507 (0.433 - 0.574)	0.364 (0.279 - 0.443)
**Combined Model**	0.549 (0.487 - 0.605)	0.596 (0.538 - 0.648)	0.590 (0.525 - 0.649)	0.461 (0.382 - 0.532)
**MammaPrint**				
**Pathologic Model**	-0.650 (-0.696 - -0.598)	-0.601 (-0.653 - -0.544)	-0.624 (-0.738 - -0.476)	-0.523 (-0.661 - -0.352)
**Clinical Model (from NCDB)**	-0.524 (-0.583 - -0.460)	-0.488 (-0.550 - -0.420)	-0.543 (-0.676 - -0.375)	-0.501 (-0.644 - -0.325)
**Combined Model**	-0.678 (-0.721 - -0.629)	-0.626 (-0.675 - -0.572)	-0.665 (-0.768 - -0.528)	-0.580 (-0.705 - -0.421)

Supplementary Table 5. Predictive Accuracy in Racial Subgroups. Results are listed for AUROC and AUPRC for prediction of high-risk recurrence score in the UCMC validation dataset with 95% confidence intervals for White (n = 288) and Black (n = 102), demonstrating that performance is preserved in racial subgroups. Other subgroups were not examined given small sample sizes limiting AUROC analysis. Expected AUPRC given random chance would be 0.167 and 0.255 for the White and Black subgroups respectively. **Abbreviations: UCMC = University of Chicago Medical Center. AUROC = Area Under the Receiver Operating Characteristic Curve. AUPRC = Area Under the Precision Recall Curve.**

	White Race (n = 288)			Black Race (n = 102)		
	**AUROC**	**AUPRC**	**p – value (AUROC compared to Combined Model)**	**AUROC**	**AUPRC**	**p – value (AUROC compared to Combined Model)**
**Pathologic Model**	0.778 (0.709 - 0.846)	0.426 (0.288 - 0.565)	0.45	0.828 (0.740 - 0.917)	0.613 (0.421 - 0.792)	0.38
**Clinical Nomogram**	0.731 (0.635 - 0.827)	0.501 (0.354 - 0.647)	0.006	0.791 (0.691 - 0.892)	0.503 (0.329 - 0.708)	0.03
**Combined Model**	0.800 (0.723 - 0.878)	0.538 (0.382 - 0.685)		0.858 (0.777 - 0.939)	0.666 (0.465 - 0.841)	

Supplementary Table 6. Select Sensitivity Analyses. We performed several sensitivity analyses to assess the robustness of our findings for prediction of the OncotypeDx score. First, we repeated training using tiles from TCGA (n = 1,039) predicted to be tumor by our tumor likelihood module, rather than using pathologist annotations. Second, we repeated training on only HR+/HER2- patients from TCGA (n = 535). Finally, we trained models on the UCMC cohort (n = 427) and validated these models in the HR+/HER2- patients in TCGA (n = 535). Of note, the combined model performance was consistently the highest and the recurrence assay could be consistently predicted above random chance in all analyses. However, performance of the pathologic model (and thus the combined model) declined when reducing the training dataset size.

	**AUROC (Training)**	**AUROC (Validation)**
**Training on TCGA Samples Filtered Using Tumor Likelihood Model**		
**Pathologic Model**	0.786 (0.675 – 0.883)	0.793 (0.739 – 0.848)
**Clinical Nomogram**	0.779 (0.645 – 0.889)	0.764 (0.697 – 0.832)
**Combined Model**	0.813 (0.695 – 0.902)	0.822 (0.766 – 0.878)
**Training Pathologic Model on Only HR+/HER2- Samples from TCGA**		
**Pathologic Model**	0.793 (0.684 – 0.896)	0.746 (0.688 – 0.804)
**Clinical Model (from NCDB)**	0.779 (0.645 – 0.889)	0.764 (0.697 – 0.832)
**Combined Model**	0.825 (0.719 – 0.907)	0.809 (0.751 – 0.868)
**Training Models on the UCMC Cohort, and Validating on TCGA**		
**Pathologic Model**	0.712 (0.557 – 0.842)	0.654 (0.587 – 0.722)
**Clinical Model (from NCDB)**	0.765 (0.618 – 0.889)	0.782 (0.724 – 0.841)
**Combined Model**	0.794 (0.652 – 0.903)	0.783 (0.726 – 0.840)

Supplementary Table 7. Prognostic Value of Models in the Training Cohort. Results are listed for Cox proportional hazard models using the specified variable as the only input, for patients receiving endocrine therapy alone and for the whole dataset. Hazard ratios are computed per standard deviation of input data given the different scales of the various models. **Abbreviations: TCGA = The Cancer Genome Atlas. CI = Confidence Interval. SD = Standard Deviation. C-Index = Concordance Index.**

	TCGA Cohort (n = 535)			
	**Hazard Ratio (95% CI)**	**z-statistic**	**p-value**	**C-Index**
	**Recurrence-Free Interval**			
**Pathologic Model**	1.10 (0.87 - 1.38)	0.8	0.422	0.549
**Clinical Nomogram**	1.33 (1.06 - 1.66)	2.5	0.012	0.644
**Combined Model**	1.14 (0.92 - 1.42)	1.23	0.217	0.606
	**Recurrence-Free Survival**			
**Pathologic Model**	1.03 (0.85 - 1.25)	0.27	0.785	0.539
**Clinical Nomogram**	1.17 (0.96 - 1.42)	1.56	0.118	0.623
**Combined Model**	1.06 (0.87 - 1.29)	0.6	0.548	0.598
	**Overall Survival**			
**Pathologic Model**	1.08 (0.86 - 1.36)	0.68	0.498	0.58
**Clinical Nomogram**	1.01 (0.8 - 1.29)	0.12	0.906	0.594
**Combined Model**	1.05 (0.83 - 1.32)	0.4	0.691	0.616

Supplementary Table 8. Utility of Models as Rule-Out Tests. Using TCGA HR+/HER2- patient data (n = 535), thresholds were averaged over threefold cross validation to yield sensitivities of 95% for high-risk recurrence scores (with linear interpolation to achieve exact result of 95%). These thresholds were applied to the UCMC dataset to evaluate performance characteristics, and assess accuracy for prediction of recurrence (measured as hazard ratio for recurrence-free interval). **Abbreviations: TCGA = The Cancer Genome Atlas. UCMC = University of Chicago Medical Center. Sen = Sensitivity. Spe = Specificity. PPV = Positive Predictive Value. NPV = Negative Predictive Value. CI = Confidence Interval.**

	TCGA				UCMC					
	**Sen (%)**	**Spe (%)**	**PPV (%)**	**NPV (%)**	**Sen (%)**	**Spe (%)**	**PPV (%)**	**NPV (%)**	**Hazard Ratio (95% CI)**	**Hazard Ratio, No Chemo (95% CI)**
**Pathologic Model**	95	53.4	24.5	98.4	78.5	64.1	33.2	92.9	2.32 (0.79 - 6.78)	3.33 (0.83 - 13.33)
**Clinical Nomogram**	95	28.5	17.4	97.3	88.6	22.1	20.5	89.5	1.15 (0.32 - 4.06)	1.21 (0.25 - 5.81)
**Combined Model**	95	44.4	21.6	98.2	87.3	55.2	30.7	95	3.73 (1.05 - 13.24)	9.94 (1.24 - 79.5)

Supplementary Table 9. Pathologic Characteristics of Predicted High Recurrence Score Patients. In slides from HR+/HER2- patients from The Cancer Genome Atlas, model predictions for high recurrence score were correlated with known pathologic features.

Feature	n Slides	t-statistic	p-value
Necrosis	559	8.518	1.52E-16
Lymphovascular Invasion	551	2.515	0.012193
Grade 3 (versus grade 2 or 1)	573	-12.411	1.76E-31
Tubule Formation (<10 % vs ≥ 10%)	561	-3.595	0.000352
Nuclear Pleomorphism (3 vs 2 & 1)	561	-11.652	2.69E-28
Mitotic Count (>10 vs ≤10)	558	-9.681	1.37E-20

Supplementary Table 10. Hyperparameters Selected for Model Training. Hyperparameters for recurrence score prediction are the result of Bayesian optimization for highest average tile area under the receiver operating characteristic curve over 50 iterations. Default hyperparameters established from training in other datasets were used for tumor identification.

Hyperparameter	Recurrence Score Prediction	Tumor Identification
Augmentations	Flip, Rotate	Flip, Rotate, JPEG compression, Blur
Batch Size	55	128
Dropout	0.31155932	0.5
Epochs	1	1
Hidden Layer Width	267	256
Hidden Layers	1	3
L1 Loss	0	0
L1 Loss (Dense Layers)	0.008306567	0
L2 Loss	0.034253246	0.00001
L2 Loss (Dense Layers)	0.039803477	0.00001
Learning Rate	0.000999282	0.0001
Learning Rate Decay	0.210307662	0.97
Learning Rate Decay Steps	1023	100000
Loss	Mean Squared Error	Sparse Categorical Crossentropy
Model	Xception	Xception
Normalizer	Reinhard	Reinhard
Optimizer	Adam	Adam
Pooling	Average	Average
Tile Pixels	299	299
Tile um	302	302
