# Supplementary material for: Integration of clinical features and deep learning on pathology for the prediction of breast cancer recurrence assays and risk of recurrence
Source: NPJ Breast Cancer. 2023 Apr 14;9:25. doi: 10.1038/s41523-023-00530-5 (PMC10104799; doi:10.1038/s41523-023-00530-5)
Supplement: Supplementary file 2 — Reporting Summary [file 41523_2023_530_MOESM2_ESM.pdf]

nature portfolio

Corresponding author(s): Alexander T. Pearson

Last updated by author(s): 3/22/2023

Reporting Summary

Nature Portfolio wishes to improve the reproducibility of the work that we publish. This form provides structure for consistency and transparency in reporting. For further information on Nature Portfolio policies, see our Editorial Policies and the Editorial Policy Checklist.

Statistics

For all statistical analyses, confirm that the following items are present in the figure legend, table legend, main text, or Methods section.

n/a	Confirmed	
☐	☒	The exact sample size (*n*) for each experimental group/condition, given as a discrete number and unit of measurement
☐	☒	A statement on whether measurements were taken from distinct samples or whether the same sample was measured repeatedly
☐	☒	The statistical test(s) used AND whether they are one- or two-sided<br>*Only common tests should be described solely by name; describe more complex techniques in the Methods section.*
☐	☒	A description of all covariates tested
☐	☒	A description of any assumptions or corrections, such as tests of normality and adjustment for multiple comparisons
☐	☒	A full description of the statistical parameters including central tendency (e.g. means) or other basic estimates (e.g. regression coefficient) AND variation (e.g. standard deviation) or associated estimates of uncertainty (e.g. confidence intervals)
☐	☒	For null hypothesis testing, the test statistic (e.g. *F*, *t*, *r*) with confidence intervals, effect sizes, degrees of freedom and *P* value noted<br>*Give P values as exact values whenever suitable.*
☐	☒	For Bayesian analysis, information on the choice of priors and Markov chain Monte Carlo settings
☐	☒	For hierarchical and complex designs, identification of the appropriate level for tests and full reporting of outcomes
☐	☒	Estimates of effect sizes (e.g. Cohen's *d*, Pearson's *r*), indicating how they were calculated

Our web collection on statistics for biologists contains articles on many of the points above.

Software and code

Policy information about availability of computer code

Data collection	Data collection was performed using Microsoft Excel 2016
Data analysis	All statistical analysis was performed in Python 3.8, Lifelines 0.27.0, and Scipy 1.8.0

For manuscripts utilizing custom algorithms or software that are central to the research but not yet described in published literature, software must be made available to editors and reviewers. We strongly encourage code deposition in a community repository (e.g. GitHub). See the Nature Portfolio guidelines for submitting code & software for further information.

Data

Policy information about availability of data

All manuscripts must include a data availability statement. This statement should provide the following information, where applicable:

- Accession codes, unique identifiers, or web links for publicly available datasets
- A description of any restrictions on data availability
- For clinical datasets or third party data, please ensure that the statement adheres to our policy

Data from TCGA including digital histology and the clinical and genetic annotations used are available from https://portal.gdc.cancer.gov/ and https://cbioportal.org, and the annotations used for grade, necrosis, and lymphovascular invasion are from previously published work. The NCDB PUF is a HIPAA-compliant data file, which is made available to investigators from CoC-accredited cancer programs who complete an application process. Trained models evaluated in this paper, anonymized patient annotations, and the complete set of tile images used for model validation can be obtained at doi.org/10.5281/zenodo.7490381.

Human research participants

Policy information about studies involving human research participants and Sex and Gender in Research.

Reporting on sex and gender	The results predominantly apply to patients of the female sex, but sex / gender were not used as a selection criteria for the study. Of 1,039 patients from The Cancer Genome Atlas included in the study, 1,027 were female sex and 12 were male sex. Of 427 patients included in our OncotypeDx validation cohort, 423 were female sex and 4 were male sex; and of the 88 patients in our MammaPrint validation cohort, all were female sex. No data was collected on self reported gender. No analysis was performed based on sex / gender given the small number of male patients.
Population characteristics	The average age of the patients from The Cancer Genome Atlas was 58.6 (standard deviation 13.2), the average age for the OncotypeDx validation cohort was 56.3 (standard deviation 10.6), and the average age for the MammaPrint validation cohort was 54.0 (standard deviation 12.7). All patients in all cohorts had a histologic diagnosis of invasive breast carcinoma.
Recruitment	Participants were selected from retrospective chart review to identify consecutive cases from January 1st 2006 through December 21st 2020 where recurrence score testing was performed and pathology was available.
Ethics oversight	University of Chicago Institutional Review Board

Note that full information on the approval of the study protocol must also be provided in the manuscript.

Field-specific reporting

Please select the one below that is the best fit for your research. If you are not sure, read the appropriate sections before making your selection.

☒ Life sciences ☐ Behavioural & social sciences ☐ Ecological, evolutionary & environmental sciences

For a reference copy of the document with all sections, see nature.com/documents/nr-reporting-summary-flat.pdf

Life sciences study design

All studies must disclose on these points even when the disclosure is negative.

Sample size	To determine the sample size needed for validation of the DL-based prediction of OncotypeDx (ODX), we stipulated a power of 80%, one-sided alpha of 0.05, an expected high-risk ODX fraction of 0.22, and an expected area under the receiver operating characteristic curve (AUROC) of 0.83 for our model combining DL on pathology and clinical characteristics, as described in our preliminary results from The Cancer Genome Atlas (TCGA). Using the methodology described by Obuchowski and a conservative null hypothesis of AUROC of 0.70, we would need a total of 34 patients with high-risk ODX scores and thus a total of 189 patients. Our validation cohort was larger than this and so was adequately powered for the study.
Data exclusions	While compiling the validation cohort, review of digitized slides identified 32 samples without identifiable tumor. These patients were excluded from analysis as our model would not be applicable to analyze pathology samples without visible tumor. This was not a pre-established exclusion criteria.
Replication	Predictions from the saved models from TCGA were replicated in duplicate and identical.
Randomization	Allocation to study groups was not random. We selected The Cancer Genome Atlas (n = 1,039) as a training dataset for our model given the larger size of this dataset, and used samples from The University of Chicago (n = 427 for OncotypeDx and n = 88 for MammaPrint) as an external validation cohort.
Blinding	As there was no group allocation, no blinding to the allocation was performed.

Reporting for specific materials, systems and methods

We require information from authors about some types of materials, experimental systems and methods used in many studies. Here, indicate whether each material, system or method listed is relevant to your study. If you are not sure if a list item applies to your research, read the appropriate section before selecting a response.

March 2021

Materials & experimental systems

n/a	Involved in the study
☒	☐ Antibodies
☒	☐ Eukaryotic cell lines
☒	☐ Palaeontology and archaeology
☒	☐ Animals and other organisms
☒	☐ Clinical data
☒	☐ Dual use research of concern

Methods

n/a	Involved in the study
☒	☐ ChIP-seq
☒	☐ Flow cytometry
☒	☐ MRI-based neuroimaging
